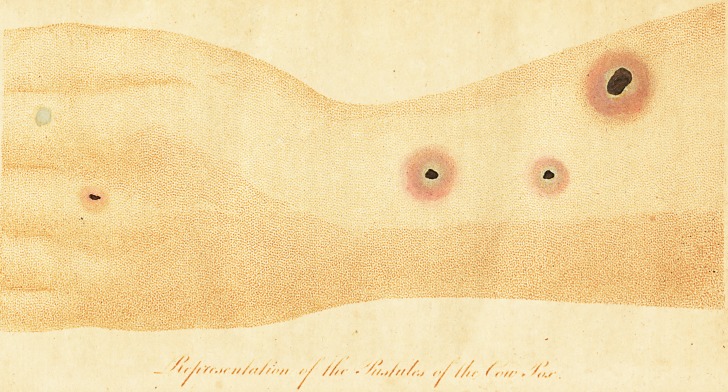# An Account of the Publications and Experiments on the Cow-Pox; Illustrated by a Coloured Plate, Which Exhibits the Appearance of the Eruption on the Arm of a Milker on the Tenth Day of the Disease. The Pock on the Forefinger Represents the Appearance of a Vesicle before Suppuration Begins

**Published:** 1799-03

**Authors:** 


### Medical and Physical Journal.

VOL. I.] FOR MARCH, 1799. [NO. I.

### *An Account of the Publications and Experiments on the Cow-pox; illuſtrated by a coloured Plate, which exhibits the appearance of the Eruption on the arm of a Milker on the tenth day of the Diſeaſe. The Pock on the Forefinger repreſents the appearance of a Veſicle before ſuppuration begins.* By *Dr.* BRADLEY.

TO trace the origin of diſeaſes which at preſent can only be propagated by contagion; or to ſuperſede one diſeaſe by inducing another, would by our forefathers have been deemed problems, the ſolution of which was too difficult to be attempted by human abilities. Our cotemporaries think otherwiſe, and perſuade themſelves that they have done more than *attempt* the ſolution. From the firſt appearance of the *ſmall-pox*, it has with reaſon been conſidered, in its natural progreſs, as one of the moſt formidable of the frequent maladies to which human beings are expoſed. SYDENHAM, by the judicious manner of treating it which he introduced, contributed greatly to diminiſh its ravages; and the practice of inoculation has ſo far reduced the number of its victims*, that where it can be employed, the ſmall-pox is ſcarcely ſo much dreaded, as the meaſles or ſcarlet fever.

There are ſome particular families, however, in which even the inoculated ſmall-pox ſeldom fails to prove a formidable diſeaſe; and we are ſorry to obſerve, that in many parts of this iſland, the prejudice againſt inoculation, eſpecially among the lower ranks of people, is far from being ſubdued; nay even many perſons in the higher claſſes are by no means convinced, when inoculation is employed in private families, that the diſeaſe is completely under medical controul. Many are perſuaded that the ſmall-pox, even when mild, not only changes the texture of the ſkin and injures the complexion, but alſo calls the latent ſeeds of ſtruma into action. If, therefore, any means could be diſcovered of ſuperſeding it without incurring equal danger, it might naturally be expected that many parents would be deſirous of taking advantage of them. This appeared to be the caſe, in

* Some are of opinion that inoculation, by keeping the small-pox always epidemic, has increased its fatality; but this conclusion appears to be drawn from insufficient premises.

in a very unequivocal manner, on the firſt publication of Dr. JENNER's work on the cow-pox, in June, 1798; for not only many perſons of diſtinction, but ſeveral medical men who rank high in the profeſſion, immediately diſcovered an earneſt deſire to have their children inoculated with the cow-pox matter as ſoon as poſſible, from the evidence of ſafety and efficacy in preventing the ſmall-pox, which was contained in Dr. Jenner's book alone. This evidence we ſhall proceed to lay before our readers. But it is proper, in the firſt place, to obſerve, that although Dr. Jenner does not claim the diſcovery of the cow-pox or the effects of it, in preventing the ſmall-pox, when taken naturally, yet he is undoubtedly intitled to all the merit and honour of having brought the ſubject of inoculating it, completely before the public, and directed the attention of the profeſſion, in general, to an inveſtigation of its truth and importance.

Dr. Jenner's book is a ſmall quarto of ſeventy-five pages, illuſtrated by four coloured plates, repreſenting the appearance of the cow-pox in the human ſubject: price 7s. 6d. Publiſhed in London, by LAW.

The author introduces his "*Enquiry into the cauſes and effects of the Cow-pox,*" by obſerving that the deviation of man from the ſtate in which he was originally placed by nature, ſeems to have proved to him a prolific ſource of diſeaſes. From the love of ſplendor, from the indulgences of luxury, and from his fondneſs for amuſement, he has familiarized himſelf with a great number of animals, which may not originally have been intended for his aſſociates.

Thoſe domeſticated animals, he obſerves, do not always affect the human race directly, as rabid ones often do; but ſometimes they affect each other primarily, and the modified diſeaſe becomes capable of producing a ſpecific action on man in a ſecondary way, which the original could not have produced. This is exemplified in what farriers term the *greaſe* in the heels of horſes, the matter of which applied to the cow produces the cow-pox, "and this is capable of generating a diſeaſe in the human body, which bears ſo ſtrong a reſemblance to the ſmall-pox, that I think it highly probable it may be the ſource of that diſeaſe." p. 2.

The matter of greaſe is applied to cows by men who have the care of horſes, being employed to aſſiſt the maid ſervants in milking. "The diſeaſe is thus communicated to the cows, and from the cows to the dairy-maids, which ſpreads through the farm, until moſt of the cattle and domeſtics feel its unpleaſant conſequences.

The

The author thus defcribes the

### Symptoms in the Cow.

" Irregular puftules appear on the nipples of the cows, which at their firft appearance are commonly of a palifh blue, or rather of a colour fomewhat approaching to livid, and are furrounded by an eryfipelatous inflammation."

Thefe puftules, unlefs a timely remedy be applied, frequently degenerate into phagedenic ulcers, which prove extremely troublefome.

The animals become indifpofed, and the fecretion of milk is much leffened.

### Symptoms in the Human Subject.

While the cows are in the ftate laft mentioned, inflamed fpots begin to appear on different parts of the hands of the domeftics employed in milking, and fometimes on the wrifts, which quickly run on to fuppuration, firft affuming the appearance of the fmall vefications produced by a burn.

Moft commonly they appear about the joints of the fingers, and at their extremities; but whatever parts are affected, if the fituation will admit, thefe fuperficial fuppurations put on a circular form, with their edges more elevated than their centre, and of a colour diftantly approaching to blue.

Abforption takes place, and tumours appear in each axilla. The fyftem becomes affected; the pulfe is quickened; and fhiverings, general laffitude, and pains about the loins and limbs, with vomitings, fupervene.

The head is painful, and the patient is now and then even affected with delirium. Thefe fymptoms, varying in their degrees of violence, generally continue from one day to three or four, leaving ulcerated fores about the hands, which, from the fenfibility of the parts, are very troublefome, and commonly heal flowly, frequently becoming phagedenic, like thofe from whence they fprung.

The lips, noftrils, eye-lids, and other parts of the body, are fometimes affected with fores; but thefe evidently arife from their being needlefsly rubbed or fcratched with the patient's infected fingers. No eruptions on the fkin have followed the decline of the feverifh fymptoms in any inftance that has come under my infpection, one only excepted, and in this cafe a very few appeared on the arms; they were very minute, of a vivid red colour, and foon died away, without advancing to maturation; fo that I cannot determine whether they had any connection with the preceding fymptoms. Thus the difeafe makes its progrefs from the horfe to the nipple of the cow, and from the cow to the human fubject. What renders the cow-pox virus fo extremely fingular is, that the perfon who has been thus affected is for ever after secure from the infection of the small-pox. In support of so extraordinary a fact, I shall lay before my reader a great number of instances."—Page 7.

Dr. Jenner then details twenty-three cases, tending to prove the foregoing opinions respecting the origin of the cow-pox, and the impossibility of the small-pox following it, provided the patient had the symptomatic fever *during the cow-pox.*

Dr. Jenner assures his readers, that the utmost care was taken to ascertain, with the most scrupulous precision, that no one whose case he adduces, had gone through the small-pox previous to his experiments; and as he lived in a part of the kingdom where population is comparatively thin, no risk of inaccuracy in this particular can arise.

In several of the cases adduced by Dr. Jenner, the patients had laboured under the cow-pox upwards of thirty years previous to the time when inoculated with variolous matter, in order to shew that the change produced in the constitution by the former is not less permanent than that by the latter.

Some of these cases are intended to prove, that persons who have had the small-pox are susceptible of the cow-pox only in a slight degree.—Page 17, &c.

At p. 21, the author observes, that although the cow-pox shields the constitution from the small-pox, and the small-pox proves a protection agaimst its own future poison, yet it appears that the human body, as well as cows, are again and again susceptible of the infectious matter of the cow-pox, as appears by the history he there adduces.

At p. 27, a case is related, where a farrier had taken a disease from the heels of a horse, and could not afterwards be infected by variolous matter. The author takes this occasion to observe, that smiths, who in the country are also farriers, are more difficultly infected by inoculation than other people; and asks, "Shall we not be able now to account for this on a rational principle?" He concludes, however, immediately after, that the disease derived from the horse is a very inferior security to that from the cow.

After several cases, tending to evince the similarity between the eruption and indisposition, as well as power of perpetuating the disease, following the insertion of vaccine and variolous matter, Dr. Jenner, at p. 41, mentions the application of a mild caustic, composed of equal parts of quick-lime and soap, to the inoculated part, in order to diminish the erysipelatous inflammation, which often takes place upon the arm, soon after the patient has sickened. He informs us, that it effectually answered his intention, in preventing the appearance appearance of eryfipelas. Indeed, it feemed to do more, for in half an hour after its application, the indifpofition of the children ceafed. What effect would a fimilar treatment produce in inoculation for the fmall-pox?

Dr. Jenner concludes his inquiry with fome general obfervations on the certainty of the inferences which he has drawn from his cafes, his own obfervations, and thofe of his friends. He confiders them as demonftrated, as far as fuch inferences can be demonftrated by an individual, whofe experiments have been exclufively confined to one part of the kingdom.

We think it both natural and defirable, that all thofe who attempt to overturn eftablifhed opinions, fhould be fomewhat more fanguine than the generality of their readers. This enthufiafm feems neceffary to procure attention, as well as to excite oppofition.

Dr. Jenner does not think the cow-pox communicable from one perfon to another by any other means than by inoculation of the matter (p. 68). He hints, that fome chronic complaints may probably receive relief from the febrile attack of cow-pox, which is *never attended with danger.*

" Thus far," fays Dr. Jenner, " have I proceeded in an inquiry, founded, as it muft appear, on the bafis of experiment; in which, however, conjecture has been occafionally admitted, in order to prefent to perfons well-fituated for fuch difcuffions, objects for a more minute inveftigation. In the mean time, I fhall myfelf continue to profecute this inquiry, encouraged by the hope of its becoming effentially beneficial to mankind."

The next work on this fubject which demands our attention, is an 8vo. pamphlet of 116 pages, by Dr. PEARSON, Nov. 1798, intituled, " *An Inquiry concerning the Hiftory of the Cow-pox, principally with a view to fuperfede and extinguifh the Small-pox.*" publifhed by JOHNSON.

This inquiry is prefaced by Dr. Pearfon with the following among other obfervations, refpecting his motives for publifhing at that time. See p. 1, 2, 3.

" In the work juft fpoken of, (Dr. Jenner's) feveral facts are related, which feem to let new light into the nature of the animal œconomy, and to exhibit a near profpect of moft important benefits in the practice of phyfic. But as fome of thefe facts do not accord, nay, as they are at variance in effential particulars with thofe to which they are neareft related, the truth of them is rather invalidated than confirmed by analogy; hence the teftimony of a fingle obferver, however experienced, and worthy to be credited, it is apprehended, is infufficient for procuring fuch facts a general acceptance. But granting that the facts fhould be generally admitted without hefitation to to be true, in the above work, the more judicious part of the medical profeffion will require the obfervations to be derived from much more extenfive and varied experience, in order to appreciate juftly, the value of the practical conclufions. Hence there appears little likelihood of improvements in practice being made, unlefs the fubject be inveftigated by many inquirers, and the attention of the public at large be kept excited. Agreeably to the preceding reprefentation, I go forward to examine the evidence of the principal facts afferted in the publication on the cow-pox ; and to ftate what farther evidence I have derived from my own experience, and from the communication of a number of profeffional gentlemen, of unfufpected veracity, and undoubted accuracy."

" Perhaps it may be right to declare, that I entertain not the moft diftant expectation of participating the fmalleft fhare of honour, on the fcore of difcovery of facts. The honour on this account, by the jufteft title, belongs exclufively to Dr. Jenner; and I would not pluck a fprig of laurel from the wreath that decorates his brow."

To the above ftatement of Dr. Pearfon's motives for publifhing, we think it right to add thofe confiderations contained in Dr. Jenner's letter to Dr. Pearfon, dated 27th Sep. 1798.

" My dear Sir,—the perufal of your proof fheets has afforded me great pleafure, both from the handfome manner in which you mention my name, and from the mafs of evidence which has poured in upon you from different countries, in fupport of the facts which I fo ardently wifh to fee eftablifhed on a fteady and durable bafis." p. 99.

In order to direct the public attention to the moft important objects of inveftigation, as well as to place them in the ftrongeft light, Dr. Pearfon has comprifed the principal facts in a feries of propofitions, to each of which he fubjoins all the information which he had then received, both from his own obfervation and perfonal inquiries, as well as the communications of his numerous correfpondents : we fhall lay the fubftance of thefe propofitions before our readers, without adhering to the precife words.— The firft propofition is to this effect. See p. 4.

1. *The cow-pox communicated in the accidental or natural way,* (*i. e.* from the teats of the cow to the hands of the milkers,) *renders the perfons who experience the fpecific fever, &c. of that difeafe, incapable of ever receiving the fmall-pox.*

To the facts adduced by Dr. Jenner, in fupport of this important propofition, from p. 9, to 26, Dr. Pearfon adds a great number of others from p. 5 to 37, not one of all which militates in the fmalleft degree againft it. This uniform confent, derived from fuch a variety of fources, will probably with many, be deemed equivalent to demonftration. Dr. Pearfon however, does not think any practical fact of fuch importance fufficiently eftablifhed, till we have at leaft a thoufand well attefted cafes in favour of it.

From the communications contained in this part of the work, as well as feveral others, it appears that the cow-pox is only known in a comparatively few counties of this ifland.

2. The fecond propofition, p. 37. "*The cow-pox communicated by* INOCULATION *renders the perfons who are affected with the fpecific fever, and peculiar local difeafe, unfufceptible of the fmall-pox.*"

This propofition involves the important fact, which, we all agree with Dr. Jenner, in wifhing to fee eftablifhed on a fteady and durable bafis.

To the cafes adduced by Dr. Jenner, Dr. Pearfon has added a confiderable number of others, from p. 37 to 42; and we can affure our readers that every week is now teeming with experiments calculated to reduce this matter to a certainty. When the refult of thefe can be fufficiently afcertained, we fhall lofe no time in laying it before the public.

3. *The matter of the cow-pox, whether taken from the brute or human body, produces the fame diforder by inoculation, and with the fame certainty; and when feveral perfons have been inoculated from each other in fucceffion, fuch removal from the original fource of the matter, produces no change in the nature or appearance of the difeafe.*

This appears to be only a branch of, or appendix to, the fecond propofition; for unlefs thefe conditions are alfo proved to be true, it is obvious that the means here intended to be recommended for fuperfeding or extinguifhing the fmall-pox can never be extenfively employed. We are therefore forry to add, this important fact at prefent is only fupported by the inftances related by Dr. Jenner.

No experiments have yet been publifhed which prove that the difeafe may be communicated by *inoculation* from the human fubject to the cow; and Dr. Pearfon adds "I apprehend that the cow-pox is the only example at prefent known, of a permanent fpecific infectious difeafe in the human conftitution, produced by matter from a different fpecies of animal; but it has often been conjectured, that many of the infectious difeafes of the human fpecies are derived from brutes."

Dr. Jenner fuggefts a caution refpecting the *ftate* of the matter employed in inoculating, and thinks, that after it has loft its limpid quality, its fpecific effect effects alfo ceafe. "Much precaution, fays he, is therefore neceffary in the progrefs of the inquiry; and this is my grand fear, that the difcovery may fall into difcredit from a want of that attention, in conducting the experiments, which the fubject requires. For example, a perfon may conceive he has the cow-pox matter on his lancet, when in fact, there may be only a little putrid pus;—with this he inoculates, and excites a difeafe of fome kind, but not fuch a one as will prevent the fmall pox. Thus a delufive inference would be drawn, at once hurtful to the caufe, and particularly injurious to me. However, truth muft appear at laft, and from your refearches its appearance will certainly be expected," See letter to Dr. Pearfon.

4. *The cow-pox may occur in the fame man or brute repeatedly, if the matter of it be applied to them, though both are equally unfufceptible of the fmall-pox.* p. 43.

This propofition appears to be received with general fcepticifm by the profeffion, merely on account of its improbability. But, furely, improbability is lighter than a feather, when put into the ballance againft fact and obfervation. All the circumftances refpecting the cow-pox, contained in the foregoing propofitions are no lefs improbable than this. Dr. Pearfon fupports the fact both by arguments and additional examples, from p. 44 to 48.

5. *A perfon who has been affected with the fmall-pox, may neverthelefs take the cow-pox.*

The evidence in favour of this propofition, is not at prefent decifive, p. 49.

6. *The cow-pox cannot be communicated by any other means than by the actual contact of the matter of a puftule.*

Some contagions appear to be conveyed in the form of gafeous fluids, as thofe of intermittents, fcarlet fever, meafles, hooping-cough. &c. Other morbific poifons are communicated, both in the ftate of effluvia, and in a palpable or vifible form, as the variolous poifon, the murrain in cattle, &c. Others again in the palpable or vifible form only as the hydrophobic, fyphilitic, and now may be added, the poifon of the cow-pox. The proofs of this propofition p. 51, 52, appear fatisfactory.

Thus far the means of communicating the cow-pox to the human fpecies and of perpetuating it there, as well as the amount of the proofs of the poffibility of fuperfeding the fmall-pox, by the introduction of it, have been fubmitted to our readers. The next queftion which demands our attention is,

7. *Is the cow-pox a fhorter, fafer, or pleafanter difeafe, than the innoculated fmall-pox, when conducted in the moft approved manner?*

Unlefs

Unlefs the affirmative of this queftion be made out to the fatisfaction of the public in general, we conclude that the fubject of this inquiry muft be deemed rather cuious than ufeful; as few parents would be found willing to expofe their children to a fevere difeafe, for the purpofe of avoiding a mild one.

Dr. Jenner and Dr. Pearfon appear to be convinced that the cow-pox is the milder and fafer of the two, as no inftance has ever occured of a perfon dying or even being in danger from it.

On the other hand, fome of Dr. Pearfon's correfpondents feem to think that the cow-pox is far more fevere than the inoculated fmall-pox. Mr. Drew obferves, p. 54, that the inoculated fmall-pox is incomparably milder than the cow-pox. Mr. Fewfter fays, p. 102. "I think it is a much more fevere difeafe, in general, than the inoculated fmall-pox. I do not fee any great advantage from inoculating for the cow-pox. Inoculation for the fmall-pox feems to be fo well underftood, that there is very little need of a fubftitute. It is curious however, and may lead to other improvements." Thefe gentlemen appear to have taken up their opinion from a few fevere cafes, and probably fome in which the ulceration on the arms, commonly noticed, was peculiarly diftreffing, as they take no notice of the *fymptomatic* fever, or any difagreable confequences of the cow-pox. The arguments on the other fide we have briefly propofed in our introduction, and a principal one is contained in the following propofitions, viz.

8. *The cow-pox never excites or predifpofes to other difeafes, which the fmall-pox has too frequently been obferved to do.*

The evidence in fupport of this important propofition, which is only a fupplement to the laft, is at prefent defective for want of time and numbers, p. 59.

9. *The cow-pox does not prevent the fmall-pox, unlefs the conftitution be affected with fever &c. during the difeafe.*

If the refult of this inquiry fhould be in favour of the cow-pox, and the public in general fhould become as defirous of adopting it to prevent the fmall-pox, as the city of London appeared to be laft fummer, it will be neceffary, that the diagnoftic fymptoms of the cow-pox fhould be accurately laid down; and that all danger of confounding it with the chicken-pox, or other cutaneous eruptions, fhould be prevented.

After the account of the evidence on the fubject of thefe propofitions, which Dr. Pearfon could obtain at that time, he proceeds to make fome obfervations on the general ftate of the queftion.

He thinks, as the information has been derived from ſo great a variety of ſources, no ſuſpicion of colluſion can ariſe; and that the plea of the ancient ſceptic, in defence of his incredulity, cannot be applied in this caſe. He was ſhewn on the walls of a temple, the votive tablets of thoſe who had eſcaped ſhipwreck in conſequence of the protection of certain deities, to whom they had addreſſed their vows, and then aſked, if he could any longer doubt the power of thoſe Gods? he ſaid, firſt let me ſee the tablets of thoſe who *periſhed* after they had implored the protection of the ſame Gods.

Since the natural ſmall-pox is known to be very often fatal; and the inoculated not oftener than once in a thouſand inſtances, as appears from the reports of Dr. Woodville and others: and ſince the *natural* cow-pox has *never* proved fatal, and the inoculated cow-pox is as much milder than the natural, as the inoculated ſmall-pox is milder than the natural; and when it is conſidered that the cow-pox is not propagated in the ſtate of effluvia; we may reaſonably conclude. "that there is great probability of the cow-pox either not proving fatal at all, or at moſt being much leſs frequently ſo, than the inoculated ſmall-pox." p. 68.

Independent of the above general inference, Dr. Pearſon thinks the "compariſon of the two diſeaſes ſhould be inſtituted with reſpect to danger under the particular circumſtances of pregnancy; age; teething; peculiar morbid ſtates; Idioſyncras; and certain epidemical ſtates."

After a comparative eſtimate of both diſeaſes, in all theſe reſpects, Dr. Pearſon continues ſtedfaſt in his adherence to the above concluſions.

Concerning the *aetiology* of the cow-pox, Dr. Pearſon concludes againſt Dr. Jenner, that it is not derived from the greaſe in horſes, nor does he believe that ſmiths or farriers are more difficultly infected by variolous matter than other people. p. 84.

Dr. Pearſon next ſuggeſts a number of other advantages, which may hereafter be derived from a ſkilful employment of the cow-pox, ſuch as the ſuſpending for a time, and thence often preventing or curing, other epidemic or contagious diſeaſes beſides the ſmall-pox, ſuch as the influenza, hooping-cough, angina maligna, &c.

Our Author concludes his work with a ſet of queries "deſigned to guide obſervation in the acquiſition of facts belonging to the ſubject of this inquiry." The moſt important of theſe are contained in the preceding propoſitions, and for the others we muſt refer our readers to the book itſelf.

The above ſhort ſketch will enable our readers to form an idea of thoſe two works, which, whatever ſuperſtructure may hereafter be raiſed, muſt always be conſidered as having layed the foundation of it.

In

In order to continue the hiſtory of the ſubject down to the preſent time, we announce to our readers, that, about the latter end of December laſt, the cow-pox broke out among the herds of ſeveral milk-farms in the environs of London. The diſeaſe ſpread rapidly, ſo that at one farm, in the ſecond and third weeks of the following month, viz. January, more than 200 out of about 850 cows were then affected, or had lately paſſed through the diſorder. At another farm, between 60 and 70 cows out of about 350 had the diſeaſe. This epizootic contagion diſappeared rapidly after the time laſt mentioned, for, by the 4th of February, not a ſingle cow could be found in ſuch a ſtate as to afford matter for inoculation. The cow-pox in this inſtance appears to have been very mild, for no loſs was experienced by the farmers from the deficiency of milk, as uſually happens.

At one of theſe farms two milkers only contracted the diſeaſe, and were affected very ſlightly; at the other farm only one out of 200 milkers was infected. A number of philoſophical and medical gentlemen, the Preſident of the royal ſociety, and the board of agriculture &c. viſited one of the above farms, to obſerve the phœnomena of the cow-pox, both among the cows and the milkers.

A ſufficient quantity of matter was collected, and a number of perſons have been inoculated of the age of two weeks and upwards. They all took the diſeaſe, and paſſed through it without being ſo ill as to be confined a ſingle day; and indeed very few of the patients made any complaint.

## Figures and Tables

**Figure f1:**